# Parsing Genetic and Autoimmune Etiology in Premature Ovarian Insufficiency

**DOI:** 10.1210/jcemcr/luad124

**Published:** 2023-12-01

**Authors:** Elise Nauwynck, Jean De Schepper, Michel De Vos, Willem Staels

**Affiliations:** Division of Pediatric Endocrinology, KidZ Health Castle, UZ Brussel, Vrije Universiteit Brussel, Brussels 1000, Belgium; Division of Pediatric Endocrinology, KidZ Health Castle, UZ Brussel, Vrije Universiteit Brussel, Brussels 1000, Belgium; Brussels IVF + Follicle Biology Laboratory (FOBI), Vrije Universiteit Brussel, Brussels 1000, Belgium; Division of Pediatric Endocrinology, KidZ Health Castle, UZ Brussel, Vrije Universiteit Brussel, Brussels 1000, Belgium; Beta Cell Neogenesis (BENE) Research Group, Vrije Universiteit Brussel (VUB), Brussels 1000, Belgium

**Keywords:** primary amenorrhea, adolescent, premature ovarian insufficiency, BMP15, FMR1

## Abstract

Premature ovarian insufficiency (POI) is a rare cause of primary amenorrhea in adolescents. For young women with uncertain etiology of POI, genetic and autoimmune testing may be recommended to assist in treatment and management decisions. This report presents a case of POI in a 16-year-old adolescent with both poly-autoimmune disease and a heterozygous missense variant in the bone morphogenic factor 15 (*BMP15*) gene, both potentially involved in the pathogenesis of POI. Accurately distinguishing between autoimmune and genetic causes is crucial for effective treatment and counseling. In addition, given the significant psychological impact and the need for reproductive options counseling, a multidisciplinary approach that includes psychological support is highly recommended.

## Introduction

Primary amenorrhea is the absence of menarche by the age of 15 years, with normal growth and secondary sexual characteristics. Premature ovarian insufficiency (POI) is a rare cause of primary amenorrhea and is defined as amenorrhea for more than 4 months before the age of 40 years, with pregnancy excluded, and at least 2 blood draws during the follicular phase revealing elevated gonadotropins, hypoestrogenism, and low serum levels of antimüllerian hormone (AMH) [1]. A comprehensive investigation of potential contributing factors is required when POI is identified. In adolescents with a normal chromosomal analysis, this investigation should include infectious, autoimmune, iatrogenic, or metabolic causes [1, 2]. Autoimmunity, based on organ-specific or non-organ-specific autoantibodies, has been regarded as a frequent cause of POI, but recent data show that the prevalence is only ∼5% [2]. Additionally, genetic testing has led to identifying more single-gene variants implicated in POI, accounting for at least 30% of cases. Pathogenic genetic defects can be located on the X chromosome and autosomes [2, 3]. Because of the wide range of candidate genes, gene panels are recommended for testing. These panels should include genes involved in the TGF-beta superfamily, such as *BMP15* and growth differentiation factor (*GDF9*). *BMP15* is an oocyte-specific growth and differentiation factor that inhibits the action of FSH, leading to follicular growth while preventing premature luteinization [3]. Heterozygous mutations in BMP15 can cause POI because of haploinsufficiency or dominant negative effects by altered protein processing, leading to a significantly reduced production and biological effect of the mature protein. In women with BMP15 variants, the age of POI onset is usually in the third decade, and approximately 80% of them have sonographic evidence of atrophic ovaries [4].

This report describes a 16-year-old girl who presented with primary amenorrhea because of POI caused by a known pathogenic heterozygous missense mutation of *BMP15* but who also had a poly-autoimmune disease. This case highlights the importance of genetic testing in idiopathic POI, even in young girls with normal-sized ovaries on pelvic ultrasound, negative family history, and evidence of poly-autoimmunity on initial screening.

## Case Presentation

A 16-year-old girl presented to our pediatric endocrine clinic with primary amenorrhea and hyperprolactinemia. She had breast development for 2 years but had no symptoms of galactorrhea, hirsutism, hot flushes, or mood swings. She had not experienced recent weight changes or monthly abdominal pain. Her medical history was unremarkable, and she was not taking any medication or supplements. Additionally, her family history was negative for autoimmune or endocrine disorders, early menopause, or fragile X syndrome.

## Diagnostic Assessment

On physical examination, her weight was 50 kg (*Z* score −0.84), height 171 cm (*Z* score 0.67), and blood pressure 130/94 mm Hg. Control reading at a second consultation showed a normal blood pressure of 112/64 mm Hg. She was at Tanner IV stage for breast and pubic hair development. Palpation of the thyroid gland was normal. Skin pigmentation and nails were normal, and she had no dysmorphic features.

Previous hormonal analysis showed elevated serum prolactin levels, high-normal FSH levels, normal LH and estradiol levels, and normal thyroid function. Repeat laboratory testing confirmed elevated prolactin with normal monomeric recovery, as well as elevated morning cortisol, high FSH, high-normal LH and SHBG, with undetectable levels of estradiol and AMH, indicating incipient POI (Table 1). The patient's karyotype, comparative genomic hybridization microarray, and fluorescence in situ hybridization of chromosome X were normal. Analysis of *FMR1* triplet repeats revealed a heterozygous intermediate expansion allele with 52 repeats. The POI gene panel (Table 2) identified a heterozygous pR68W mutation in *BMP15*. Screening for antiovarian, antiadrenocortical, and anti-21-hydroxylase antibodies was repeatedly negative, but antithyroid peroxidase (TPO) and antiparietal cell antibodies were consistently elevated (Table 3). Hematological examination revealed normal erythrocyte sedimentation rate and white blood cell count but iron deficiency anemia. Serum gastrin was elevated, whereas tissue transglutaminase IgA and fecal occult blood tests were negative. Endoscopic-histologic examination confirmed the presence of atrophic gastritis. Sequencing of the autoimmune regulator (*AIRE)* gene was normal. Abdominal ultrasonography revealed normal-sized ovaries without antral follicles and a uterus with size 3.8 × 1.6 × 3.3 cm and endometrial thickness of 5 mm. Magnetic resonance imaging of the pelvis confirmed normal ovarian size and showed 1 fluid-filled clear cyst of 9 mm in diameter in the right ovary. Magnetic resonance imaging of the brain showed a hypointense lesion (2.8 mm in diameter) in the posterior part of the anterior pituitary. Bone mineral density of the spine was normal (dual energy X-ray absorptiometry, *Z*-score of −0.2).

**Table 1.** Hormonal and hematologic data

Variable	Values	Normal range
**HORMONES**		
TSH	3.9mIU/L (3.9mIU/L)	0.27-4.2mIU/L (0.27-4.2mIU/L)
FT4	0.97ng/dL (12.5pmol/L)	0.85-1.86ng/dL (11.0-24.0pmol/L)
LH	13.6IU/L (13.6IU/L)	0.53-41.7IU/L (0.53-41.7IU/L)
FSH	23.3IU/L (23.3IU/L)	1.6-17.0IU/L (1.6-17.0IU/L)
Prolactin	56.2ng/ml (56.2ng/ml)	3.71-23.12ng/ml (3.71-23.12ng/ml)
Prolactin after monomeric recovery	66.2% (66.2%)	60-100% (60-100%)
Cortisol	18.6µg/dL (516nmol/L)	2.7-10.4µg/dL (75-287nmol/L)
17 OH progesterone	1.5µg/L (4.54nmol/L)	0.21-1.4µg/L (0.64-4.24nmol/L)
Estradiol	<5ng/L (<18pmol/L)	18.4-247ng/L (67.5-906.8pmol/L)
Progesteron	0.35µg/L (1.11nmol/L)	
Testosteron	0.24µg/L (0.83nmol/L)	
SHBG (nmol/, (µg/L)	159µg/L (1.67nmol/L)	9.3-75.2µg/L (0.1-0.8nmol/L)
AMH (pmol/L, ng/mL)	<0.03ng/mL (<0.2pmol/L)	0.66-8.4ng/mL (4.7-60pmol/L)
**HEMATOLOGY**		
Red blood cells ()	4.8 x10E^6^/mm^3^ (4.8 x10E^6^/mm^3^)	3.9-5.0 x10E^6^/mm^3^ (3.9-5.0 x10E^6^/mm^3^)
Hemoglobin (g/L, g/dL)	9.1g/dL (0.9g/L)	11.8-14.5g/dL (1.2-1.5g/L)
Hematocrit (L/L, %)	29.4% (0.29L/L)	36.4-43.9% (0.36-0.44L/L)
Mean cellular volume (fL)	60.7fL (60.7fL)	83.0-98.0fL (83.0-98.0fL)
Iron (µmol/L, µg/dL)	16µg/dL (2.9µmol/L)	25-107µg/dL (4.5-19.2µmol/L)
Ferritin (pmol/L, µg/L)	3µg/L (6.7pmol/L)	7-140µg/L (15.7-314.6pmol/L)

Abbreviation: AMH, antimüllerian hormone.

**Table 2.** Included genes in POI gene panel

*AFF2*	ALF transcription elongation factor 2
*AMH*	Antimullerian hormone
*AMHR2*	Antimullerian hormone receptor 2
*BMP15*	Bone morphogenic factor 15
*DACH2*	Dachshund family transcription factor 2
*DAZL*	Deleted in azoospermia
*DIAPH2*	Diaphanous related formin 2
*DMC1*	DNA meiotic recombinase 1
*ESR1*	Estrogen receptor 1
*FIGLA*	Factor in the germline alpha
*FMR1*	Fragile X mental retardation
*FOXL2*	Forkhead box L2
*FOXO1*	Forkhead box protein O1
*FOXO3*	Forkhead box protein O3
*FSHR*	FSH receptor
*GDF9*	Growth differentiation factor 9
*GPR3*	G-protein coupled receptor 3
*HFM1*	Helicase for meiose 1
*INHA*	Inhibin subunit alpha
*LHCGR*	LH/human chorionic gonadotrophin receptor
*MCM8*	Mini-chromosome maintenance proteins 8
*MSH5*	MutS homolog 5
*NOBOX*	NOBOX oogenesis homeobox
*NR5A1*	Nuclear receptor subfamily 5 group A member 1
*PATL2*	PAT1 homolog 2
*PGRMC1*	Progesterone Receptor Membrane Component 1
*POF1B*	POF1B actin binding protein
*SOHLH1*	Spermatogenesis and oogenesis specific basic helix-loop-helix 1
*SOHLH2*	Spermatogenesis and oogenesis specific basic helix-loop-helix 2
*STAG3*	Stromal antigen 3
*TGFBR3*	TGF Beta Receptor 3
*XPNPEP2*	X-prolyl aminopeptidase 2

**Table 3.** Immunological screening

Variable	Values	Normal range
**IMMUNOGLOBULINS**		
IgG	12.13g/L (80.9µmol/L)	5.50-14.4g/L (36.7-96.1µmol/L)
IgA	1.12g/L (7µmol/L)	0.61-3.48g/L (3.8-21.8µmol/L)
IgM	1.74g/L (1.74µmol/L)	0.26-2.32g/L (0.26-2.32µmol/L)
**AUTO-ANTIBODIES**		
Antinuclear antibodies	Negative	Negative
Antiovarian antibodies	Negative	Negative
Thyroperoxidase antibodies	>600kIU/L	<34kIU/L
Parietal cell antibodies	1/2560	<1/40
Adrenocortical antibodies	1/0	<1/5
21-hydroxylase antibodies	<4U/ml (<4U/ml)	<4U/ml (<4U/ml)
Tissue-transglutaminase IgA antibodies	0.5U/ml (0.5U/ml)	<10U/ml (<10U/ml)
Deamidated gliadin antibodies	0.8U/ml (0.8U/ml)	<10U/ml (<10U/ml)

## Treatment

After receiving a single dose of IV iron to treat her iron-deficiency anemia, she was prescribed oral supplementation. Despite being diagnosed with POI and having undetectable levels of AMH, she refused hormone-replacement therapy. Because of her low AMH levels, temporary corticoid treatment or oocyte cryopreservation were not offered as options.

## Outcome and Follow-up

One year after diagnosis, hormonal analysis showed persistently elevated FSH (22.1 U/L) and LH (9.8 U/L), with unmeasurable serum AMH. Anti-TPO and antiparietal cell antibodies remained positive, but thyroid and adrenal function were normal.

## Discussion

We present a rare case of an adolescent girl with POI caused by a heterozygous mutation in *BMP15*, accompanied by poly-autoimmune disease. The clinical presentation of POI in adolescents depends on the age of onset. If ovarian follicular depletion occurs during childhood, it can result in growth retardation and a lack of pubertal maturation. In later stages, patients may present with arrested puberty, abnormal uterine bleeding because of anovulatory cycles, or primary or secondary amenorrhea [1]. Our patient presented with primary amenorrhea, normal secondary sexual characteristics, and no other symptoms, which raised suspicion for POI.

Initially, autoimmune POI was considered the most likely diagnosis in our patient for several reasons. First, she had multiple autoantibodies, including anti-TPO and antiparietal cell antibodies. Thyrogastric syndrome is a well-known autoimmune association, with a prevalence of 5% for thyroid autoimmune diseases and 2% for autoimmune atrophic gastritis. This association is due to a combination of a common embryological origin, namely the endoderm, and a common genetic susceptibility, namely HLA class II [5]. Although atrophic autoimmune gastritis is commonly associated with Hashimoto thyroiditis, Addison adrenalitis, or type 1 diabetes, the association with autoimmune POI has not yet been described in the literature.

Second, autoimmune POI can present without antisteroidogenic or antiovarian antibodies, which means that their absence in our patient does not necessarily rule out the diagnosis [6]. Furthermore, normal-sized ovaries with residual follicular structures, as seen in our patient, are often seen on ultrasound in the early stages of autoimmune POI, whereas atrophy is only seen in the final stage [7]. Given the suspicion of autoimmune oophoritis and the presence of several autoantibodies, a search for an underlying *AIRE* mutation was undertaken. Recently, autosomal dominant heterozygous *AIRE* gene mutations have been associated with milder manifestations than classical autoimmune polyendocrine syndrome type 1, including only POI and hypothyroidism in the absence of chronic mucocutaneous candidiasis [8]. However, a gene panel approach led to the discovery of an established pathogenic *BMP15* mutation.

The etiology of POI includes a significant genetic component, accounting for 7% to 30% of cases. Candidate genes involved in primordial germ cell migration and proliferation, cell death (*FMR1*), oocyte-specific transcription, folliculogenesis, TGF-beta superfamily (*BMP15* and *GDF9*), and hormone reception have been identified. Among the most common genetic abnormalities associated with POI are *FMR1* premutations and 45,X or 45,X/46,XX karyotypes, and *BMP15* gene defects [3].

Fragile X syndrome is a form of X-linked intellectual disability caused by more than 200 CGG repeats (full mutation) in the 5′ untranslated region of the *FMR1* gene. Premutation alleles ranging from 59 to 199 repeats have a high probability of expanding to more than 200 repeats in 1 generation and can cause Fragile X-associated POI with the development of POI resulting from an mRNA toxic gain-of-function mechanism. Intermediate alleles ranging from 45 to 54 CGGs, as in our case, can become unstable when transmitted from parent to child, leading to a full mutation in several generations. Studies examining the possible influence of intermediate *FMR1* CGG expansions on POI manifestation have produced varying results because of different definitions of intermediate alleles, small study sizes, and publication bias [9]. The repeat size, the role of AGG interspersions that generally occur every 10 CGG repeats, the mean age at menopause of first-degree relatives, ethnicity, and interactions with other genes or environmental factors may play a role in this association [9, 10].

*BMP15* is an oocyte-specific growth/differentiation factor belonging to the TGF-beta superfamily, which plays a crucial role in follicle formation and granulosa cell growth. It is located on Xp11.2, a critical chromosomal region for ovarian differentiation. After proteolytic processing, BMP15 peptides either form noncovalently linked homodimers or bind to GDF9 to transform into their biologically active form, known as cumulin. BMP15 primarily inhibits the actions of FSH by directly suppressing the mRNA expression of FSH receptors in granulosa cells. As a result, the suppression of FSH-responsive messages leads to the inhibition of FSH-induced progesterone production. Loss of BMP15 activity can result in defective follicle proliferation in the presence of higher FSH levels and precocious luteinization. Over the past few decades, several pathogenic variants in the *BMP15* gene have been identified, mainly in the heterozygous state and located in the pro-region [3]. The heterozygous mutations can cause POI, probably because of haploinsufficiency or dominant negative effects resulting from partial functional loss of the mutant protein from altered protein processing, misfolding, defective secretion, reduced protein stability, and impaired bioactivity on the target granulosa cells [4]. Because of the cleavage of pro-region during BMP15 maturation, bioinformatics analyses are likely to underestimate the effects of these mutations as the prodomain remains actively involved in the homo- and heterodimers secretion and function. Most carriers of these mutations were diagnosed with POI before age 30 years and had atrophic ovaries on ultrasound [3]. The presentation of our subject is unique because POI occurred at the early age of 16 years and the patient had normal-sized ovaries. The p.R68W mutation present in our patient has been repeatedly reported in studies screening large cohorts of women with POI. This mutation leads to a marked reduction in the production and biological effect of mature BMP15 [3].

Regarding the treatment of POI, estrogen replacement therapy is essential to either induce puberty or preserve sexual characteristics, adequate bone mineral accumulation, and prevent long-term neurological, metabolic, and cardiovascular consequences of estrogen deficiency. Unlike typical menopause, POI differs in that ovarian failure may be transient, and spontaneous pregnancy is reported in 5% to 10% of diagnosed POI cases because of improved ovarian function on FSH normalization with hormone-replacement therapy. Ovarian cortex cryopreservation may be an option for girls diagnosed with POI early before serum AMH levels become undetectable and complete ovarian follicle depletion sets in [2].

This case illustrates the value of genetic investigations in young females with POI, even in the presence of autoantibodies. The diagnosis of X-linked dominant forms of POI is significant for genetic counseling because it facilitates the screening of family members. This, in turn, allows for timely intervention through hormone replacement therapy to mitigate associated comorbidities or the option of egg cryopreservation to optimize future fertility prospects.

## Learning Points

- Genetic defects are a significant cause of premature ovarian insufficiency (POI) and may be found in adolescents with suspected autoimmune oophoritis.
- In cases of idiopathic POI, genetic screening for mutations in *FMR1* and *BMP15* should be performed.
- POI caused by a *BMP15* mutation can present during adolescence with normal-sized ovaries at ultrasound.

## Data Availability

Data sharing does not apply to this article as no datasets were generated or analyzed during the current study.

## Abbreviations

AMH: antimüllerian hormone
POI: premature ovarian insufficiency
TPO: thyroid peroxidase
